# Metagenomic and Transcriptomic Analyses Reveal the Differences and Associations Between the Gut Microbiome and Muscular Genes in Angus and Chinese Simmental Cattle

**DOI:** 10.3389/fmicb.2022.815915

**Published:** 2022-04-05

**Authors:** Ya Zheng, Juanjuan Chen, Xiaoxuan Wang, Ling Han, Yayuan Yang, Qi Wang, Qunli Yu

**Affiliations:** ^1^College of Food Science and Engineering, Gansu Agricultural University, Lanzhou, China; ^2^Cuiying Biomedical Research Center, Lanzhou University Second Hospital, Lanzhou, China; ^3^Gansu YaSheng Hiosbon Food Group Co., Ltd., Lanzhou, China

**Keywords:** Angus and Chinese Simmental cattle, compositional and functional differences, differentially expressed genes, gut microbiome, meat quality

## Abstract

Gut microbiome and heredity are two important factors affecting the intramuscular fat (IMF) of cattle, excluding age, sex, and nutrition. This study aimed at deciphering these two differences by analyzing the gut microbiome and intramuscular differentially expressed genes (DEGs) in the Angus and Chinese Simmental cattle. Feces and longissimus dorsi were collected from the two groups of animals (*n* = 20/group) for multiomics analysis. Angus holds a significantly higher diversity than Chinese Simmental, and the relative abundance of *Roseburia*, *Prevotella*, *Coprococcus*, etc., was obviously higher in Angus. Chinese Simmental had higher levels of isobutyrate, isovalerate, and valerate, although similar levels of acetate, propionate, and butyrate were observed for the two groups. The DEGs upregulated in Chinese Simmental were mainly involved in immune and inflammatory responses, while those in Angus were associated with the regulation of muscle system and myofibril. We finally identified 17 species, including *Eubacterium rectale*, etc., which were positively correlated to muscle and fat metabolism genes (*MSTN*, *MYLPF*, *TNNT3*, and *FABP3/4*) and illustrate the associations between them. Our study unveils the gut microbial differences and significant DEGs as well as their associations between the two breeds, providing valuable guidance for future mechanism research and development of intervention strategies to improve meat quality.

## Introduction

The Angus cattle in China are imported purebred cattle from Scotland in recent years and mainly used for beef production for their large muscle content with superior meat quality. The Chinese Simmental cattle is a cross from the Simmental cattle in Switzerland and the Chinese native cattle, with a history about 50 years, which is versatile for milk and meat production. Angus beef meat is superior in juiciness, tenderness, and flavor to Chinese Simmental for higher intramuscular fat (IMF), which makes it more popular and marketable. As a direct indicator of meat quality, IMF is influenced by a variety of factors, including the hosts’ age, sex, nutrition, and genetics. The *AKIRIN2*, *TTN*, *EDG1*, and *MYBPC1* genes are well-known marbling-related genes in Japanese black beef cattle and Chinese Qinchuan cattle (Li et al., 2020). The steroid biosynthesis and peroxisome proliferator-activated receptor (PPAR) signaling pathway are reported for lipid storage and metabolism in broiler chickens (Liu et al., 2019). Proteins related to glycolysis or gluconeogenesis, oxidative metabolism, and slow-type muscle or retinoic acid metabolism were the most abundant in the high-adiposity cattle group (Bazile et al., 2019). Hitherto, the beef gene expression of the Angus and Chinese Simmental cattle is unclear. In addition, as a newly discovered factor of IMF and muscular mass and function, the gut microbiome is reported in recent years in mice (Lahiri et al., 2019), chickens (Wen et al., 2019), pigs (Qi et al., 2019; Chen et al., 2021), and humans (Liu et al., 2021). As a ruminant animal, the rumen microbiota has been studied in recent years, and *Ruminococcus*, *Butyrivibrio*, *Coprococcus*, *Shuttleworthia*, *Prevotella*, and *Treponema* have been found as the prominent microbiota in cattle (Xue et al., 2018). However, few studies on the gut microbiota in Angus and Chinese Simmental cattle have been reported, not to mention the correlation between the gut microbiota and muscular gene expression.

The gut microbiota and their metabolites have played important roles in IMF deposition by affecting lipid metabolism (Schoeler and Caesar, 2019), which might be mediated by short-chain fatty acids (SCFAs) *via* the following mechanisms: (1) used as an additional energy source for animals and affect energy metabolism (Ley et al., 2006); (2) acted as signaling molecules to modulate GPR43 and GPR41 genes in white adipose tissue and L-cell (McNelis et al., 2015); and (3) butyrate and propionate can activate the PPARγ signaling pathway in liver and adipose tissue (Alex et al., 2013). Besides SCFAs, bile acids (Parseus et al., 2017), lipopolysaccharides (Cani et al., 2007), trimethylamines (Meng et al., 2018; Bollatti et al., 2020), tryptophan, and their derivatives (Zelante et al., 2013) were also reported to play a role in host lipid metabolism, and the underlying mechanism needs further exploration. In addition to lipid metabolism, recent studies have also shown that decreased muscle mass and/or function were compatible with gut microbial composition changes in mice (Ni et al., 2019) and rat (Siddharth et al., 2017). The *Bacteroides fragilis* gnotobiotic mice showed higher function and muscle mass compared with germ-free (GF) mice (Donaldson et al., 2020). The microbiome-transplanted mice from different donors, including lean or obese pigs (Yan et al., 2016), healthy or malnourished children (Blanton et al., 2016), pathogen-free (PF) mice (Lahiri et al., 2019) and conventionally raised mice (Nay et al., 2019), further evidenced the relationship of muscle and gut microbiota. Given the relationship between the gut microbiome and fat metabolism and muscle mass and/or function, interventions targeting the gut microbiota were considered to improve the meat quality in meat-producing animals. *Lactobacillus* species (Lee et al., 2018), *Bacillus subtilis* H2 (Shi et al., 2020), *Prevotella copri* (Chen et al., 2021), tryptophan (Goodarzi et al., 2021), vitamin B12 (Boachie et al., 2020), folate (Mlodzik-Czyzewska et al., 2020), and SCFAs (Jiao et al., 2021) were beneficial to fat metabolism and deposition. *Saccharomyces boulardii*, *Lactobacillus casei* LC122, *Bifidobacterium longum* BL986 (Jiao et al., 2021), *Lactobacillus paracasei* PS23 (Chen et al., 2019), *Lactobacillus salivarius* SA-03 (Lee et al., 2020), *Lactobacillus plantarum* TWK10 (Chen et al., 2016), and *Bifidobacterium longum* OLP-01 (Huang et al., 2020) were reported to affect muscular mass and function.

The gut microbiome has also been implicated in host gene regulation. Human genes may influence the chemical signature by shaping the composition of the skin microbiota (Verhulst et al., 2010). In a genetically susceptible host, imbalances in microbiota–immunity interactions under defined environmental contexts are believed to contribute to the pathogenesis of a multitude of immune-mediated disorders (Zheng et al., 2020). Vagnerová et al. (2019) analyzed the influence of the microbiota on acute restraint stress response and suggested that the downregulated expression of pituitary *Pomc* and *Crhr1* in specific-pathogen-free animals might be an important factor in the exaggerated hypothalamic–adrenal response of GF mice to stress. Genes linked to inflammatory bowel disease (IBD) modulate microbial recognition and innate immune pathways (*Nod2*), and genes that mediate autophagy (i.e., *ATG16L1*, *IRGM*) have highlighted the critical role of host–microbe interactions in controlling intestinal immune homeostasis (Cucchiara et al., 2012). Chen et al. (2020) had evaluated the association of the oral microbiome with host gene methylation and patient outcomes and implied *F. nucleatum* as a pro-inflammatory driver in initiating head and neck squamous cell carcinoma. Dayama et al. (2020) characterized changes in the microbiome and host gene expression, and identified host gene–microbiome interactions in the colonic mucosa might play a role in the pathogenesis of cystic fibrosis patients. In cancer, an alteration of microbiome is thought to affect the regulation of various miRNAs through the MyD88-dependent pathway, and miRNA alteration is associated with gut dysbiosis, in turn, to affect cancer onset and prognosis (Allegra et al., 2020). Integration of multi-omic approaches, also known as holo-omics (Nyholm et al., 2020), is a new approach to incorporate multi-omic data from both host and microbiota domains to untangle the interplay between the two, which will broaden our perspective of the molecular mechanisms involved in disease (Nguyen et al., 2020). In the present study, we have combined metagenomics, transcriptomics, and targeted metabolomics to illustrate the underlying mechanism of gut microbiota on the IMF of the two cattle.

Differences on IMF of the Angus and Chinese Simmental cattle were observed (Supplementary Figure 1 and Supplementary Table 1). Here we compared the gut microbiome (*via* shotgun metagenomic sequencing), longissimus dorsi muscular gene expression (*via* RNA-seq), and SCFAs (*via* targeted metabolomics) in Angus and Chinese Simmental. Firstly, the gut microbial characteristics and DEGs between the two cattle were illustrated. Then, we identified correlations between host muscular gene expression and gut microbiome data by using an integrative analysis approach, which allowed us to characterize potential interactions between host genes and microbes, providing insight on improving meat quality by altering the gut microbiota. We have also hoped that these host gene–microbiome associations can serve as a precursor for designing future hypothesis-driven studies.

## Materials and Methods

### Animal Experiment and Sample Collection

The cattle were selected from Gansu Zhangye Qilian Muge Co., Ltd. (Zhangye, Gansu, China). Angus (*n* = 20, male, with a final average body weight of 713.36 ± 41.35 kg) and Chinese Simmental (*n* = 20, male, with a final average body weight of 687.18 ± 33.86 kg) cattle of 18 months were fed with the same diet for at least 10 months on the same farm. Rectal fecal samples were collected with a sterile spoon by cut a notch in the rectum after the cattle were slaughtered. 5 g fecal sample was collected in a pre-marked 15 ml sterile centrifuge tube and quickly move in anaerobic bag in dry ice after hanging tight the lid. The Longissimus dorsi were collected as soon as the cattle were slaughtered. 10 g muscular tissue were quickly collected and packaged in a pre-marked 2 ml sterile cryogenic vials and freeze in liquid nitrogen for RNA sequencing after the cattle were slaughtered. The collected fecal samples and tissues were delivered through dry ice to the laboratory for DNA, SCFAs and RNA extraction, concentration and purity testing, library construction and sequencing. The samples used for multiomics were shown in Supplementary Table 2A.

### DNA Extraction and Shotgun Metagenomic Sequencing

Total genomic DNA was extracted from the rectal fecal sample in accordance with the MetaHIT protocol as described previously (Qin et al., 2012). The quality and quantity of the DNA was measured using NanoDrop Spectrophotometer ND-1000 (Thermo Fisher Scientific Inc.). Metagenome library was constructed using the TruSeq DNA PCR-Free Library Preparation Kit (Illumina), and the quantity of each library was evaluated using a Qubit 2.0 fluorimeter (Invitrogen). Sequencing of metagenome libraries was conducted at BGI-Shenzhen (Shenzhen, China) using BGI-Seq500 (150bp paired-end sequencing of ∼350bp inserts) (Fang et al., 2018).

### RNA Extraction and Transcriptomic Sequencing

Total RNA was isolated from the muscular tissue of the two breeds using the Trizol Reagent (Invitrogen Life Technologies), after which the concentration, quality, and integrity were determined using a NanoDrop spectrophotometer (Thermo Scientific). Only samples with RNA integrity number ≥ 7.0 were used to generate transcriptome libraries. In the current study, mRNA-enriched transcriptome libraries were constructed. Enriched mRNA was used for transcriptome library construction using TruSeq RNA Library Prep Kit v2 (Illumina). The sequencing library was then sequenced on NovaSeq 6000 platform (Illumina) by Shanghai Personal Biotechnology Co., Ltd.

### Detection of Short-Chain Fatty Acids

#### Chemicals and Reagents

Methyl tert-butyl ether (MTBE) was purchased from CNW (CNW Technologies, Germany). MilliQ water (Millipore, Bradford, MA, United States) was used in all experiments. All of the standards were purchased from 12CNW (Beijing) or Aladdin (Shanghai). The stock solutions of standards were prepared at the concentration of 1 mg/ml in MTBE. All stock solutions were stored at −20°C. The stock solutions were diluted with MTBE to working solutions before analysis.

#### Sample Preparation and Extraction

In total, 50 mg of rectal fecal samples was accurately weighed and placed in a 2-ml Eppendorf (EP) tube. Then, 0.2 ml of phosphoric acid (0.5% v/v) solution and a small steel ball were added to the EP tube. The mixture was ground for 15 s for 3 times, then vortexed for 10 min, and ultrasonicated for 5 min. Then, 0.5 ml MTBE (containing internal standard) solution was added to the EP tube. The mixture was vortexed for 3 min and ultrasonicated for 5 min. After that, the mixture was centrifuged at 14,400 rcf for 10 min at the temperature of 4°C. The supernatant was collected and used for gas chromatography (GC)–tandem mass spectrometry (MS/MS) analysis.

#### Gas Chromatography–Tandem Mass Spectrometry Analysis

Agilent 7890B gas chromatograph coupled to a 7000D mass spectrometer with a DB-5MS column (30 m, length × 0.25 mm, i.d. × 0.25 μm, film thickness, J&W Scientific, United States) was employed for the GC–MS/MS analysis of SCFA contents. Helium was used as carrier gas at a flow rate of 1.2 ml/min. Injections were made in the splitless mode, and the injection volume was 2 μl. The oven temperature was held at 90°C for 1 min, raised to 100°C at a rate of 25°C/min, raised to 150°C at a rate of 20°C/min, held for 0.6 min, raised to 200°C at a rate of 25°C/min, and held for 0.5 min after running for 3 min. All samples were analyzed in multiple reaction monitoring mode. The injector inlet and transfer line temperature were 200 and 230°C, respectively.

### Analysis of Shotgun Metagenomics

#### Metagenomic Sequencing Data Process

Samples are sequenced on the BGI-Seq 500 platforms to get raw sequences (FASTQ format). Paired-end metagenomic sequencing was performed on the BGI-Seq500 platform with a read length of 150 bp (insert size, 300 bp). The raw reads that had 50% low-quality bases (quality ≤ 20) or more than five ambiguous bases were excluded. The remaining reads were mapped to *Bos taurus*^1^ to remove cattle genomes. The high-quality non-host sequencing reads (clean reads) were used in taxonomic annotation.

#### Taxonomic and Functional Annotation

The clean reads were used to generate taxonomic profiles by using Metaphlan 3.0 (-input_type fastq - ignore_viruses - nproc 6) as described in the official website [MetaPhlAn3-The Huttenhower Lab (harvard.edu)]. HUMAnN 3.0 (Franzosa et al., 2018) (-i input_clean_data -o output –threads 10 –memory-use maximum –remove-temp-output) was used to efficiently and accurately profile the abundance of microbial metabolic pathways and other molecular functions from the clean data according to the official website.^2^

#### Diversity Calculation

Alpha-diversity [within-sample diversity, R 4.0.3 vegan: diversity (data, index = “richness/shannon/Simpson/InSimpson”)] was calculated using the richness, Shannon index, Simpson index, and inverse Simpson index depending on the taxonomic profiles. Beta-diversity [between-sample diversity, R 4.0.3 vegan: diversity (data, index = “bray_curtis distance”)] was calculated using the Bray–Curtis distance depending on the gene and taxonomic profiles.

### Analysis of Transcriptomes

#### Quality Control

The samples are sequenced on the platforms to get image files, which are transformed by the software of the sequencing platform, and the original data in FASTQ format (raw data) is generated. The sequencing data contains a number of connectors and low-quality reads, so we use Cutadapt (v1.15) software to filter the sequencing data to get a high-quality sequence (clean data) for further analysis. Non-rDNA/rRNA reads were then mapped to the bovine genome (UMD 3.1) using Tophat2 (version 2.0.9) as described by Kim et al. (2013) to remove potential host DNA and RNA contaminations.

#### Reads Mapping

The reference genome and gene annotation files were downloaded from the genome website. The filtered reads were mapped to the reference genome using HISAT2 v2.0.5 [HISAT2 (daehwankimlab.github.io)].

#### Differential Expression Analysis

We used HTSeq (0.9.1) statistics to compare the read count values on each gene as the original expression of the gene and then used FPKM to standardize the expression. Then, difference expression of genes was analyzed by DESeq (1.30.0) with the screening conditions as follows: expression difference multiple | log2 (fold change)| > 1, significant *P*-value < 0.05. At the same time, we used R language Pheatmap (1.0.8) software package to perform bi-directional clustering analysis of all different genes of the samples. We retained the heat map according to the expression level of the same gene in different samples and the expression patterns of different genes in the same sample with the Euclidean method to calculate the distance and complete linkage method to cluster.

#### Gene Ontology and Kyoto Encyclopedia of Genes and Genomes Enrichment Analysis

We mapped all the genes to the terms in the Gene Ontology (GO) database and calculated the number of differentially enriched genes in each term. Using top GOs to perform GO enrichment analysis on the differential genes, we calculated the *P*-value by the hypergeometric distribution method (the standard of significant enrichment is *P*-value < 0.05) and found the GO term with significantly enriched differential genes to determine the main biological functions performed by differential genes. For the transcriptomes, ClusterProfiler (3.4.4) software was used to carry out the enrichment analysis of the Kyoto Encyclopedia of Genes and Genomes (KEGG) pathways of differential genes, focusing on the significantly enriched pathway with *P* < 0.05. For the metagenomes, HUMAnN 3.0 was used to generate the functional modules described as in Franzosa et al. (2018).

### Statistical Analysis

For the identified species, DEGs, functional modules, and KEGG pathways, the relative abundance of each was compared between the two breeds *via* Wilcoxon rank-sum test, followed by Benjamini and Hochberg correction. Non-parametric multivariate analysis of variance (permutational multivariate analysis of variance) was used to analyze the degree of explanation for sample differences by different grouping factors in PCoA. Wilcoxon rank-sum test was used to analyze the differences of metagenomic data between the two groups. Student’s *t*-test was used to compare the transcriptomic data between the two groups.

The correlation between the differentially enriched species (*P*-value less than 0.05 between the two groups, and the occurrence is more than 0) and the DEGs (*P*-value less than 0.05 between the two groups) between the two groups were analyzed by Spearman’s rank-sum correlation [code: R 4.0.3, cor.test (*,*,method = “spearman”)].

## Results

### The Gut Microbial Taxonomy of Angus Cattle Was Significantly Different From Simmental Cattle

**FIGURE 1:**
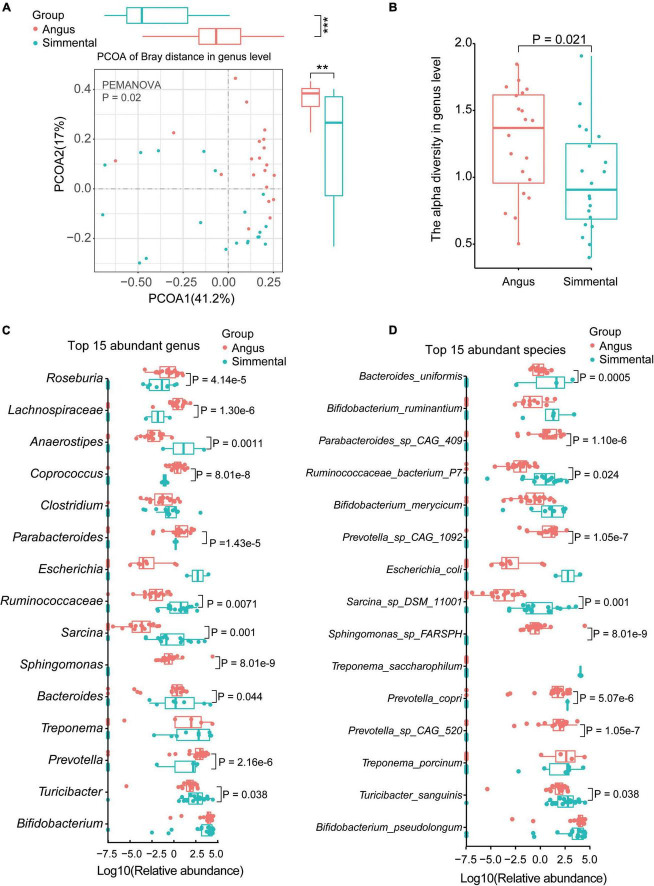
Taxonomic analysis of the gut microbiota for the Chinese Simmental and Angus cattle. The Angus cattle had higher beta-diversity **(A)** and alpha-diversity **(B)** than the Chinese Simmental cattle at genus level. The differences of the top 15 abundant genera **(C)** and species **(D)** are shown.

Ten phyla (Supplementary Table 2B), 77 genera (Supplementary Table 2C), and 179 species (Supplementary Table 2D) were annotated by MetaPhlan3.0. Significant differences of the gut microbiome were observed between the Angus cattle and the Simmental cattle. The species composition, diversity, and relative abundance of the Angus cattle were significantly higher than that of the Simmental cattle (beta-diversity, *P* = 0.001, Supplementary Figure 2A; alpha-diversity, *P* = 0.021, Supplementary Figure 2B). At the genus level, the gut microbiome also showed significant differences between and within groups (beta-diversity, *P* = 0.02; Figure 1A; alpha-diversity, *P* = 0.021, Figure 1B).

We then analyzed the top 15 abundant genera (Figure 1C), top 15 species (Figure 1D), and top 5 abundant phyla (Supplementary Figure 2C). Among the five top abundant phyla, the predominant Actinobacteria showed no significant difference between the two breeds. The relative abundance of Firmicutes (*P* = 0.02) was significantly higher in the Angus cattle, while that of Bacteroidetes (*P* = 0.0001) was obviously higher in the Simmental cattle (Supplementary Figure 2C). At the genus level, *Roseburia* (*P* = 4.14e-5), *Lachnospiraceae* (*P* = 1.30e-6), *Coprococcus* (*P* = 8.01e-8), *Parabacteroides* (*P* = 1.43e-5), *Sphingomonas* (*P* = 8.01e-9), *Bacteroides* (*P* = 8.01e-8), and *Prevotella* (*P* = 2.16e-6) were significantly higher in the Angus cattle. *Anaerostipes* (*P* = 0.0011), *Ruminococcaceae* (*P* = 0.0071), *Sarcina* (*P* = 0.001), and *Turicibacter* (*P* = 0.038) were obviously higher in the Simmental cattle. Some common genera such as *Bifidobacterium*, *Clostridium*, *Escherichia*, and *Treponema* had little differences between the two breeds. At the species level, *Parabacteroides* sp. CAG:409 (*P* = 1.10e-6), *Prevotella* sp. CAG:1092 (*P* = 1.05e-7), *Prevotella* sp. CAG:520 (*P* = 1.05e-7), and *Sphingomonas* sp. FARSPH (*P* = 8.01e-9) were significantly enriched in the Angus cattle. *Sphingomonas* sp. was thought to be a nosocomial infectious organism (Ryan and Adley, 2010). The relative abundance of *Bacteroides uniformis* (*B. uniformis*, *P* = 0.0005), *Ruminococcaceae bacterium P7* (*R. bacterium P7*, *P* = 0.024), *Sarcina* sp. DSM 11001 (*P* = 0.001), *Prevotella copri* (*P. copri*, *P* = 5.07e-6), and *Turicibacter sanguinis* (*T. sanguinis*, *P* = 0.038) was significantly higher in the Simmental cattle. Interestingly, the *Bifidobacterium* sp. (*B. ruminantium*, *B. merycicum*, and *B. pseudolongum*), *Treponema* sp. (*T. porcinum*, *T. saccharophilum*), and *Escherichia coli* (*E. coli*) showed no significant difference between the two groups. *B. pseudolongum* can reduce triglycerides by modulating the gut microbiota in mice fed high-fat food (Bo et al., 2020). *T. saccharophilum* is a large pectinolytic spirochete from the bovine rumen (Paster and Canale-Parola, 1985), and *T. porcinum* is isolated from porcine faces (Nordhoff et al., 2005).

### Functional Differences Between the Angus and Simmental Cattle

**FIGURE 2:**
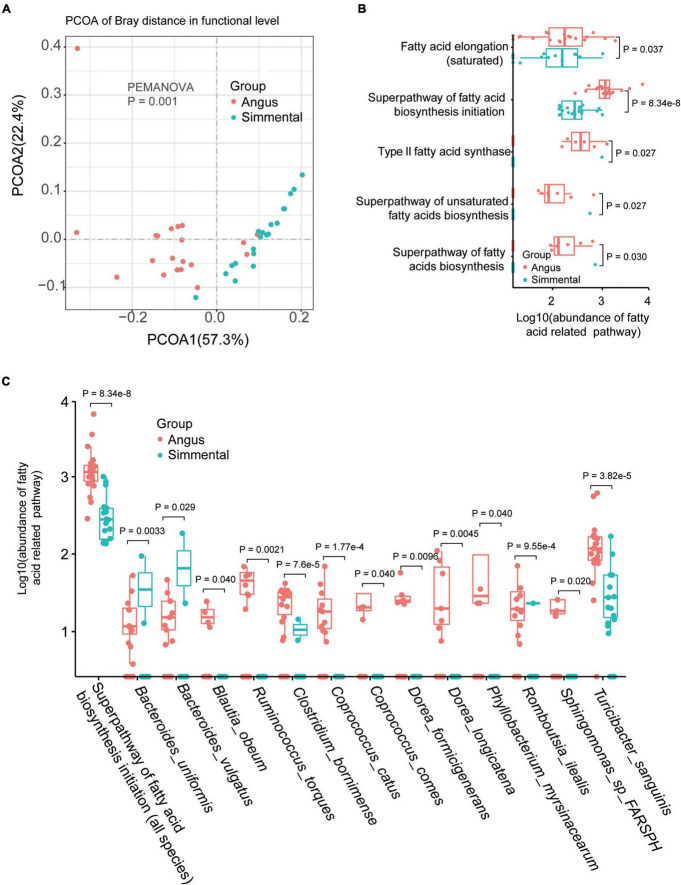
Gut microbial functional differences and the related species. Gut microbial function was significantly different between the two breeds **(A)**. The abundance of the fatty acid-related pathway was obviously different for the two breeds **(B)**. Species related to the superpathway of fatty acid biosynthesis initiation were also significantly different between the two breeds **(C)**.

Significant differences existed between the two groups (*P* = 0.001, Figure 2A). In total, 354 gut microbiome functional pathways (Supplementary Table 2F) were annotated by HUMAnN 3.0. Among them, 208 pathways were differentially enriched in the two breeds. Only 7 pathways, including stearate biosynthesis III, L-glutamate degradation VIII (to propanoate), anaerobic energy metabolism (invertebrates and cytosol), and gluconeogenesis III, were significantly highly enriched in the Simmental cattle. The superpathway of branched-chain amino acid biosynthesis, aliphatic amino acid biosynthesis, aromatic amino acid biosynthesis, glycolysis, and gluconeogenesis were all highly enriched in the Angus cattle. The superpathway of lipopolysaccharide biosynthesis showed no significant difference in the two breeds. We then focused on the fatty-acid-related pathways and found that the superpathway of fatty acid biosynthesis initiation (*P* = 8.34e-8), fatty acid elongation (*P* = 0.037), type II fatty acid synthase (*P* = 0.027), superpathway of unsaturated fatty acids biosynthesis (*P* = 0.027), and superpathway of fatty acid biosynthesis (*P* = 0.030) were all highly enriched in the Angus cattle (Figure 2B). These results were consistent with the muscular phenotypes, indicating that the Angus cattle hold more types of fatty acids than the Simmental cattle (Supplementary Table 1). We further studied the association between the gut microbiota and the superpathway of fatty acid biosynthesis initiation (Supplementary Figure 3). In total, 13 significantly related species including *B. uniformis*, *Bacteroides vulgatus* (*B. vulgatus*), *Blautia obeum* (*B. obeum*), *Ruminococcus torques*, *Clostridium bornimense*, *Coprococcus catus (C. catus)*, *Coprococcus comes*, *Dorea formicigenerans*, *Dorea longicatena*, *Phyllobacterium myrsinacearum*, *Sphingomonas* sp. FARSPH, and *T. sanguinis* were observed. Except for *B. uniformis*, *B. vulgatus*, and *Romboutsia ilealis*, all the other species were obviously abundant in the Angus cattle (Figure 2C).

### Differentially Expressed Genes in the Beeves of Angus and Simmental Cattle

**FIGURE 3:**
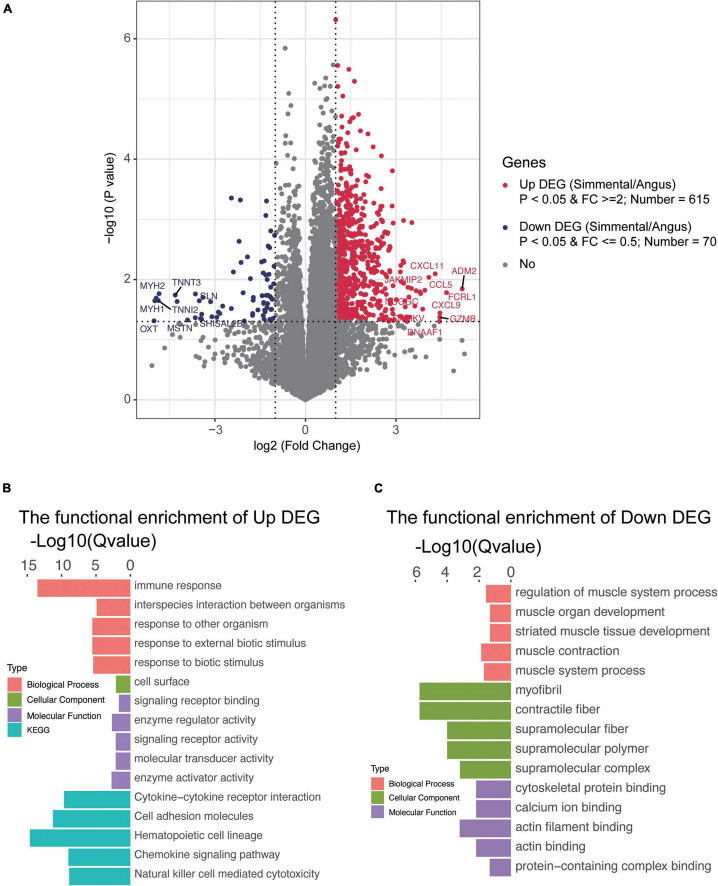
Transcriptomic analysis of the differentially expressed genes (DEGs) and their function in the muscular tissue of Angus and Chinese Simmental cattle. The ratio of Chinese Simmental cattle to Angus cattle (Simmental/Angus) meant up and down. Among the DEGs between the two breeds, 615 genes were upregulated **(A)**, and their function are shown in **(B)**; 70 DEGs were downregulated **(A)**, and their function are shown in **(C)**.

To understand the differences in gene expression between the two kinds of cattle, the longissimus dorsi was collected to perform RNA sequencing. In total, 14,345 genes were detected, and 1,254 DEGs (the fold change of the ratio for Simmental/Angus was either greater than 2 or less than 0.5) were identified between the two breeds (Figure 3A and Supplementary Table 2G). The principal component analysis plot showed that the DEGs between the two groups of cattle were different (Supplementary Figure 6). The Simmental cattle had higher mRNA levels of 615 DEGs (upregulated DEGs, *P* < 0.05) including *ADM2*, *CAMKV*, *CCL5*, *CXCL11*, *CXCL9*, *DNAAF1*, *FCRL1*, *GZMB*, *JAKMIP2*, and *NUGGC*, while the Angus cattle had higher mRNA levels of 70 DEGs (downregulated DEGs, *P* < 0.05) including *MSTN*, *MYLPF*, *TNNI2*, *TNNT3*, *MYBPC2*, *MYH1*, *MYH2*, *OXT*, *SHISAL2B*, and *SLN*. We then focused on the muscle mass and function-related genes (e.g., *MSTN*, *MYLPF*, *TNNI2*, *TNNT3*, *MYBPC2*, *ACTN3*, *ATP2A1*, and *MYL1*), and they all had higher levels in the Angus cattle. We also identified some interesting DEGs related to immune and inflammatory responses and muscular mass and function. In the muscular tissue of the Chinese Simmental cattle, genes involved in immune and inflammatory responses (e.g., *TLR7*, *CD14*, *CCL25*, *CCL27*, *CCL5*, *CCL22*, *IL1A*, *IL1RL1*, *IL2RA*, *IL2RB*, *IL2RG*, *IL7R*, *IL10RA*, *IL12A*, *IL12RB1*, *IL18RAP*, *IL21R*, *IL22RA1*, *IL27*, *JAKMIP2*, and *FCRL1*), genes involved in muscular formation (e.g., *CAMKV*, *MYO1G*, *MYO1F*, *CDKL5*, *METTL21C*, *CDKL3*, and *VDR*), and genes related to lipolysis and lipid transport (e.g., *APOL3* and *DGAT2L6*) had higher mRNA levels. On the contrary, the lipid transport gene *ADIPOQ* and lipogenesis and lipid deposition (e.g., *FABP4* and *FABP7*) had higher levels in the Angus cattle. These results suggested that the Chinese Simmental cattle had more muscular genes changed in immune and inflammatory response and muscle formation, while the Angus cattle hold more genes changed in inhibiting muscle formation (*MSTN*) and lipid transport, lipogenesis, and lipid deposition.

To understand the function of these DEGs, ClusterProfiler (3.4.4) software was used for the GO and KEGG annotations (Figures 3B,C and Supplementary Figure 5). The functional capacities of the upregulated DEGs in Chinese Simmental cattle were mainly located in the cell surface (cellular component, CC), whose molecular function (MF) mainly include signaling receptor binding, enzyme regulator activity, signaling receptor activity, molecular transducer activity, and enzyme activator activity and finally play a role in the biological process (BP), such as immune response, interspecies interaction between organisms, response to other organism, response to external biotic stimulus, and response to biotic stimulus. The KEGG items of these genes were mainly focused on cytokine–cytokine receptor interaction, cell adhesion molecules, hematopoietic cell lineage, chemokine signaling pathway, and natural killer cell-mediated cytotoxicity. Meanwhile, the function of the downregulated DEGs in Angus cattle was mainly expressed in myofibril, contractile fiber, supramolecular fiber, supramolecular polymer, and supramolecular complex (CC), whose MF mainly focus on cytoskeletal protein binding, calcium ion binding, actin filament binding, actin binding, and protein-containing complex binding and play an important role in the BP, including regulation of muscle system process, muscle organ development, striated muscle tissue development, muscle contraction, and muscle system process. The functional capacities annotated by GO and KEGG once again proved that the Angus cattle had higher capacities in regulating muscle metabolism, lipogenesis, and lipid deposition, while the Chinese Simmental cattle hold more capacities in immune and inflammatory responses, which might explain the differences in (IMF) between the two breeds.

### The Differentially Expressed Genes Were Associated With the Differential Species

**FIGURE 4:**
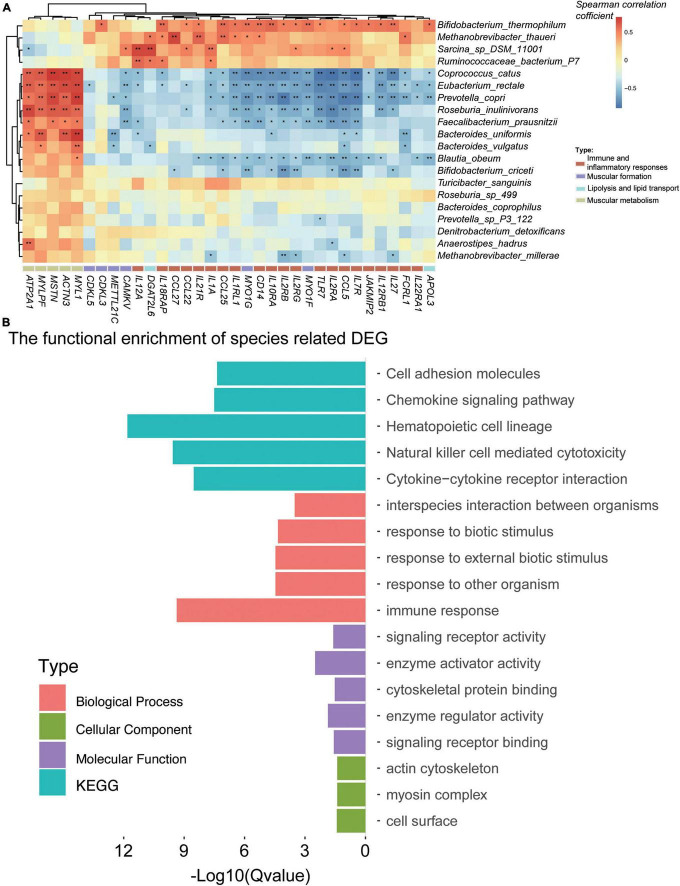
Gut microbiota was associated with the differentially expressed genes (DEGs) for the Angus and Chinese Simmental cattle. In total, 17 species were significantly (the correlation coefficient is greater than 0.6) associated with the DEGs **(A)**. Function of the DEG-related species **(B)**.

To study the association between the DEGs and the gut microbiome, the DEGs were associated with the differential species by Spearman’s rank correlation (the absolute value of the correlation coefficient is more than 0.6; Figure 4A). A total of 17 species were related to 486 DEGs (Supplementary Table 2I). We have focused on the genes in immune and inflammatory responses, muscular metabolism and formation, lipogenesis, and lipid metabolism and deposition. Interestingly, the muscular metabolism-related genes (e.g., *ATP2A1*, *MSTN*, *ACTN3*, *MYLPF*, *MYL1*, and *TNNT3*) were all positively correlated with *R. inulinivorans*, *E. rectale*, *C. catus*, *B. uniformis*, and *B. vulgatus*. It is worth mentioning that *Faecalibacterium prausnitzii* (*F. prausnitzii*) was also positively correlated with *MSTN*, *MYL1*, and *ATP2A1*. The immune and inflammatory response genes (*TLR7*, *CD14*, *CCL25*, *CCL27*, *CCL5*, *CCL22*, *IL1A*, *IL1RL1*, *IL2RA*, *IL2RB*, *IL2RG*, *IL7R*, *IL10RA*, *IL12A*, *IL12RB1*, *IL18RAP*, *IL21R*, *IL22RA1*, *IL27*, *JAKMIP2*, and *FCRL1*), muscular formation genes (*CAMKV*, *MYO1G*, *MYO1F*, *CDKL5*, *METTL21C*, *CDKL3*, and *VDR*), lipolysis and lipid transport genes (*APOL3* and *DGAT2L6*), and lipogenesis and lipid deposition genes (*FABP4* and *FABP7*) (Figure 4A) were negatively correlated with *B. uniformis*, *B. vulgatus*, *R. inulinivorans*, *E. rectale*, *C. catus*, and *F. prausnitzii*, while *Bifidobacterium thermophilum*, *Methanobrevibacter thaueri*, *Sarcina* sp. DSM 11001, and *R. bacterium* P7 were positively correlated with these DEGs.

To evaluate the functional capacities of the species-related DEGs, we have also annotated the GO and KEGG pathway of the DEGs (Figure 4B). The altered genes were mainly located in actin cytoskeleton, myosin complex, and cell surface (cellular component). The molecular function of these genes was mainly enriched in enzyme activator and regulator activity, signaling receptor activity and binding, and cytoskeletal protein binding, which might play a role in the biological process, including immune response, response to biotic stimulus/external biotic stimulus/other organisms, and interspecies interaction between organisms. The KEGG pathways of these genes mainly focused on hematopoietic cell lineage, natural killer cell-mediated cytotoxicity, cytokine–cytokine receptor interaction, chemokine signaling pathway, and cell adhesion molecules.

### Short-Chain Fatty Acids and Their Associations With the DES and Differentially Expressed Genes Between the Two Breeds

We have evaluated the SCFAs between the two breeds by targeted metabolomics on the fecal samples (Supplementary Table 2H). Among the 7 SCFAs, acetate, propionate, and butyrate, which were reported to contribute to the IMF to improve the meat quality, showed no difference between the two breeds. However, the concentration of isobutyric acid (*P* = 0.028), isovaleric acid (*P* = 0.019), valeric acid (*P* = 0.032), and caproic acid (*P* = 1.4e-4) was significantly higher in the Chinese Simmental cattle (Supplementary Figure 4).

To address the association between the SCFAs and differentially enriched species (Supplementary Figure 7) and DEGs (Supplementary Figure 8), Spearman’s rank correlation was performed. The results suggested that *C. catus*, *Eubacterium rectale*, *P. copri*, *F. prausnitzii*, *B. obeum*, *B. uniformis*, and *B. vulgatus* were all negatively associated with isobutyrate, isovalerate, valerate, and caproate, while *Sarcina* sp. DSM 11001, *R. bacterium* P7, and *Turicibacter sanguinis* showed a positive association with the above-mentioned SCFAs. For the top 3 DEGs, isobutyrate, isovalerate, valerate, and caproate were positively correlated with *NLRP3*, *BTK*, *NCF2*, *OMG*, *LRRC15*, *TDRKH*, *CXCL16*, and *IL18RAP*, while they were negatively correlated with *UCMA*, *BTBD11*, *STEAP4*, *CALY, AFF3*, *ACTN3*, *AGBL1*, and *MYL1*.

## Discussion

The IMF of the Angus and Chinese Simmental cattle is different, and the host genetics and gut microbiome have been reported as important factors for IMF. To reveal the differences on the gut microbiome and muscular gene expression as well as their relationship between the two breeds, muscular RNA-seq, rectal fecal shotgun metagenomic sequencing, and targeted metabolomics for rectal fecal SCFAs were performed in our present study.

Significant differences of the gut microbiota exist between the two breeds. The Angus cattle hold higher species, genus, and phylum diversity and richness compared to the Chinese Simmental cattle (Supplementary Table 2E). The top abundant phyla, genera, and species were also significantly different between the two breeds. The Angus cattle hold a lower Firmicutes/Bacteroidetes (F/B) ratio of 0.65, while the F/B ratio for Chinese Simmental cattle was 4.49. F/B is important in maintaining normal intestinal homeostasis, and increased F/B is usually observed with obesity (Stojanov et al., 2020), which is consistent with the phenotype which has a heavy dewlap. In addition, we should pay attention to the significantly enriched species in the Chinese Simmental cattle, such as *B. uniformis*, *P. copri*, and *T. sanguinis. B. uniformis* was reported to combine with fiber to amplify metabolic and immune benefits in obese mice (Lopez-Almela et al., 2021). *P. copri* can increase fat accumulation in pigs fed with formula diets (Chen et al., 2021). *T. sanguinis*, a novel serotonin sensor in the spore-forming gut bacteria (Hoffman and Margolis, 2020), might be important for host lipid and steroid metabolism (Fung et al., 2019). These results suggested that reducing the relative abundance of *B. uniformis* and *P. copri* while increasing the relative abundance of *T. sanguinis* might be beneficial for lipid metabolism and deposition. It is worth noting that no obvious differences have been detected in *Lactobacillus* and *Bifidobacterium*, which were thought to regulate lipid metabolism in animals fed with high-fat diet (Xie et al., 2011; Salaj et al., 2013; Lee et al., 2018) or with hyperlipidemia (Qian et al., 2019). This might be also a reason why there was no difference on SCFAs, including acetate, butyrate, and propionate production (den Besten et al., 2013). In addition, there were no significant differences on some opportunistic pathogens, including *E. coli* and *Clostridium*, between the two breeds, which meant that there was no difference in outbreaks of mass diseases between the two groups (Fancher et al., 2020).

The functional capacities of the gut microbiome were also significantly different between the two breeds. The Angus cattle had a higher capacity of fatty acid biosynthesis, which is consistent with the more types of fatty acids in their longissimus dorsi (Supplementary Table 1). It is worth mentioning that most of the gut microbial species, which were related to fatty acid biosynthesis, were also significantly highly enriched in the Angus cattle. We have also examined the SCFAs in the rectal fecal sample, and isobutyric acid, isovaleric acid, valeric acid, and caproic acid were significantly higher in the Chinese Simmental cattle, while acetate, butyrate, and propionate showed no differences between the two breeds, which further illustrated that the rectum is not the main fermenter organ for ruminant animals and that ruminal samples should be used for related studies of SCFAs (acetate, butyrate, and propionate). SCFAs, particularly acetate, propionate, and butyrate, are mainly produced by the anaerobic fermentation of gut microbes and play an important role in regulating energy metabolism and energy supply as well as maintaining the homeostasis of the intestinal environment (He et al., 2020). In addition, acetate and butyrate were major substrates for *de novo* lipogenesis in rats (Zambell et al., 2003). Valerate, isovalerate, and isobutyrate are produced exclusively by the bacterial fermentation of proteinaceous material (polypeptides and amino acids), and they are putrefactive, whose presence suggests underlying maldigestion and/or malabsorption from dysfunctional states, such as hypochlorhydria or exocrine pancreatic insufficiency, or bacterial overgrowth in the small intestine (*Isobutyric Acid—An Overview* | ScienceDirect Topics). We have also correlated the differently expressed SCFAs with differentially enriched species (Supplementary Figure 7) and top 3 DEGs (Supplementary Figure 8). The results suggested that isobutyrate, isovalerate, valerate, and caproate were positively correlated with *C. catus*, *Eubacterium rectale*, *P. copri*, *F. prausnitzii*, *B. obeum*, *B. uniformis*, and *B. vulgatus*, while they were negatively associated with *Sarcina* sp. DSM 11001, *R. bacterium* P7, and *Turicibacter sanguinis*. The GO annotations related to DEGs that were positively correlated with isobutyrate, isovalerate, valerate, and caproate were mainly including immune activity, such as *NCF2*, *BTK*, and *NLRP3*, while the function of the negatively correlated DEGs was mainly concentrated on the structural constituent of the muscle, calcium ion binding, and decarboxylating/ferric-chelate reductase/metallocarboxypeptidase activity, such as *MYL1*, *AGBL1*, *ACTN3*, *STEAP4*, etc. These results suggested that the gut microbiota might affect the muscular metabolism by SCFAs, including isobutyrate, isovalerate, valerate, and caproate, except for the generally recognized acetate, propionate, and butyrate.

The DEGs were analyzed to describe the genomic differences between the two groups. In total, 615 DEGs were significantly highly expressed in Chinese Simmental cattle, and 70 DEGs were obviously highly expressed in Angus cattle. The functional capacities of the highly expressed DEGs in the Chinese Simmental cattle mainly focused on inflammatory diseases (*CXCL9* and *FCRL1*), immunodeficient diseases (*ADM2*, *CCL5*, and *CAMKV*), carcinoma (*CXCL11*, *JAKMIP2*, *GZM*, and *DNAAF1*), and GTP binding and GTPase activity (*NUGGC*). However, in the Angus cattle, although there were genes involved in inflammatory disease (*OXT*) and microcystic adenoma (*SHISAL2B*), the main function of DEGs was enriched in arthrogryposis (*TNNT3* and *TNNI2*) and myopathy and myasthenic syndrome (*MSTN*, *MYH2*, and *SLN*). These results suggested that the muscular gene expression and their function of the Angus and Chinese Simmental cattle were significantly different, and the Chinese Simmental cattle might be superior in anti-inflammatory, immunodeficient diseases and carcinoma, while the Angus cattle were more active in muscle metabolism. In addition, some lipid metabolism genes including *ADIPOQ*, *FABP4*, and *FABP7* hold higher mRNA levels in the Angus cattle, which meant that the Angus cattle were superior in lipid transport, lipogenesis, and lipid deposition.

Finally, to reveal the relationship between the gut microbiome and the host muscular gene expression, we performed Spearman’s rank correlation analysis for the interesting DEGs and differentially enriched species between the two breeds. As expected, we have retained some gut microbial species including *B. uniformis*, *B. vulgatus*, *R. inulinivorans*, *E. rectale*, *C. catus*, and *F. prausnitzii*, which were all positively correlated with the muscular metabolism-related genes including *ATP2A1*, *MSTN*, *ACTN3*, *MYLPF*, *MYL1*, and *TNNT3.* Among these species, *R. inulinivorans*, *E. rectale*, and *C. catus* were significantly highly abundant in the Angus cattle, while *B. uniformis* and *B. vulgatus* were obviously enriched in the Chinese Simmental cattle. These results implied that supplements of *R. inulinivorans*, *E. rectale*, *C. catus*, and *F. prausnitzii* might benefit the muscular metabolism in the Chinese Simmental cattle. We have also paid attention to the DEGs which were associated with immune and inflammatory responses, muscular formation, lipolysis and lipid transport, and lipogenesis and lipid deposition (Figure 4A). Interestingly, *B. uniformis*, *B. vulgatus*, *R. inulinivorans*, *E. rectale*, *C. catus*, and *F. prausnitzii* were all negatively correlated with these DEGs, and *Bifidobacterium thermophilum*, *Methanobrevibacter thaueri*, *Sarcina* sp. DSM 11001, and *R. bacterium* P7 were positively correlated with these DEGs. *B. thermophilum* is a relatively oxygen-tolerant Bifidobacterium species that has been isolated from bovine rumen, sewage, and piglet, calf, and baby feces (Toure et al., 2003), which impacts on the growth and virulence gene expression of *Salmonella Typhimurium* (Tanner et al., 2016) and affects the gut microbiota of Göttingen minipigs (Tanner et al., 2015). *R. bacterium* P7 is also a rumen microbiota (Won et al., 2020), and its functions were not studied yet. *Methanobrevibacter thaueri* is an anaerobe, mesophilic archaeon that was isolated from bovine feces, which might have an effect on the dynamic profile of end and intermediate metabolites of anaerobic fungus *Piromyces* sp. F1 (Li et al., 2016).

To address the relationships and the underlying philosophy and methodology between host genetic variation and gut microbes, some research have been made in recent years (Wang et al., 2018). The microbes that inhabited the human and animal gastrointestinal, skin, oral respiratory, and reproductive systems can be classified as beneficial, harmful, and moderate ones. The beneficial ones can produce a large amount of nutrients and essential functional molecules and maintain the normal functions of the immune systems, and the primary role of the host genes is to ensure their immune tolerance and facilitate their growth by secreting mucus, *etc*., as substances. The harmful microbes usually produce toxins and pro-inflammatory molecules and lead to infections. The host genes must clear them from the normal community and defend against inflammation and infections (Wang et al., 2018). IBD continues to be the central focus of host–microbe investigations. Genetic studies have revealed some potential genetic risk factors in IBD patients, including *NOD2*, *CARD9*, *ATG16L1*, *IRGM*, and *FUT2* (Xavier and Podolsky, 2007), which are indeed significantly associated with the decrease in the genus *Roseburia*, who plays an essential role in the conversion of acetate to butyrate. In our study, we have found the changes of some harmful species and genes related to immune and inflammatory responses. We assumed that these species might affect the gut barrier to disturb the inflammatory cytokine secretion, thus influencing the lipid and muscular metabolism. In addition, the different levels of SCFAs (isobutyrate, isovalerate, and valerate) between the two breeds also hinted that the gut microbiota might affect the IMF by affecting the lipid metabolism and deposition by SCFA mediating. The intricate mechanism under the gut microbiota and host genes in these two cattle will be further verified in our future studies.

## Conclusion

In conclusion, our study revealed the compositional and functional differences in the gut microbiome as well as the differences in muscular gene expression and their association with the gut microbiome between the Angus and Chinese Simmental cattle of the same age, sex, and feeding style. We suggested that the addition of some beneficial gut microbiota and supplements such as *R. inulinivorans*, *E. rectale*, *C. catus*, *F. prausnitzii*, acetate, propionate, and butyrate or the reduction of other seemingly unfriendly species such as *P. copri*, *B. uniformis*, and *B. vulgatus* might improve the meat quality. However, as ruminant animals, the gastrointestinal tract is unique, and rumen is thought to be the main part for fermentation. We need to further explore the relationship between the rumen microbiota and the IMF between the two breeds in the future.

## Data Availability Statement

The authors acknowledge that the data presented in this study must be deposited and made publicly available in an acceptable repository, prior to publication. Frontiers cannot accept a manuscript that does not adhere to our open data policies.

## Ethics Statement

The animal study was reviewed and approved by GB/T 19477-2018.

## Author Contributions

QY conceived the study. QW took part in the project design and sample collection and was also in charge of data analysis and graphics presentation. YZ was in charge of sample and phenotype collection, took part in manuscript writing, and basic data analysis. JC designed and wrote the manuscript. XW determined the concentration of fatty acids in the longissimus dorsi. QY and QW modified the manuscript. All other authors have participated in the work and have read and edited the manuscript.

## Conflict of Interest

XW was employed by the Gansu YaSheng Hiosbon Food Group Co., Ltd. The remaining authors declare that the research was conducted in the absence of any commercial or financial relationships that could be construed as a potential conflict of interest.

## Publisher’s Note

All claims expressed in this article are solely those of the authors and do not necessarily represent those of their affiliated organizations, or those of the publisher, the editors and the reviewers. Any product that may be evaluated in this article, or claim that may be made by its manufacturer, is not guaranteed or endorsed by the publisher.

## Footnotes

1
http://ftp.ensembl.org/pub/release-105/fasta/bos_taurus/dna/

2
https://huttenhower.sph.harvard.edu/humann

## Abbreviations

IMF: intramuscular fat
DEGs: differentially expressed genes
*R. inulinivorans*: *Roseburia inulinivorans*
*E. rectale*: *Eubacterium rectale*
*C. catus*: *Coprococcus catus*
*P. copri*: *Prevotella copri*
*F. prausnitzii*: *Faecalibacterium prausnitzii*
*T. sanguinis*: *Turicibacter sanguinis*
*B. ruminantium*: *Bifidobacterium ruminantium*
*B. merycicum*: *Bifidobacterium ruminantium*
*B. pseudolongum*: *Bifidobacterium pseudolongum*
*T. porcinum*: *Treponema porcinum*
*T. saccharophilum*: *Treponema saccharophilum*
*E. coli*: *Escherichia coli*
SCFAs: short-chain fatty acids
Bas: bile acids
LPS: lipopolysaccharides
TMA: trimethylamines
PPARγ: peroxisome proliferator-activated receptor γ
GPR43: G protein-coupled receptor 43
GPR41: G protein-coupled receptor 41
FXR: farnesoid X receptor
TGR5: G-protein -coupled bile acid receptor
TLR4: Toll like receptor 4
GLP-1: glucagon-like peptide 1
GF: germ-free
PF: pathogen-free
RIN: RNA integrity number
MTBE: methyl tert-butyl ether
GO: Gene Ontology
KEGG: Kyoto Encyclopedia of Genes and Genomes
FC: fold change
Mrna: microRNA
CC: cellular component
MF: molecular function
BP: biological process
F/B: Firmicutes/Bacteroidetes.

## References

[B1] Alex S. Lange K. Amolo T. Grinstead J. S. Haakonsson A. K. Szalowska E. (2013). Short-chain fatty acids stimulate angiopoietin-like 4 synthesis in human colon adenocarcinoma cells by activating peroxisome proliferator-activated receptor gamma. *Mol. Cell. Biol.* 33 1303–1316. 10.1128/MCB.00858-12 23339868 PMC3624264

[B2] Allegra A. Musolino C. Tonacci A. Pioggia G. Gangemi S. (2020). Interactions between the MicroRNAs and Microbiota in Cancer Development: Roles and Therapeutic Opportunities. *Cancers* 12:805. 10.3390/cancers12040805 32230762 PMC7225936

[B3] Bazile J. Picard B. Chambon C. Valais A. Bonnet M. (2019). Pathways and biomarkers of marbling and carcass fat deposition in bovine revealed by a combination of gel-based and gel-free proteomic analyses. *Meat. Sci.* 156 146–155. 10.1016/j.meatsci.2019.05.018 31158601

[B4] Blanton L. V. Charbonneau M. R. Salih T. Barratt M. J. Venkatesh S. Ilkaveya O. (2016). Gut bacteria that prevent growth impairments transmitted by microbiota from malnourished children. *Science* 351:10. 10.1126/science.aad3311 26912898 PMC4787260

[B5] Bo T. B. Wen J. Zhao Y. C. Tian S. J. Zhang X. Y. Wang D. H. (2020). Bifidobacterium pseudolongum reduces triglycerides by modulating gut microbiota in mice fed high-fat food. *J. Steroid. Biochem. Mol. Biol.* 198:105602. 10.1016/j.jsbmb.2020.105602 31987886

[B6] Boachie J. Adaikalakoteswari A. Samavat J. Saravanan P. (2020). Low Vitamin B12 and Lipid Metabolism: Evidence from Pre-Clinical and Clinical Studies. *Nutrients* 12:1925. 10.3390/nu12071925 32610503 PMC7400011

[B7] Bollatti J. M. Zenobi M. G. Artusso N. A. Alfaro G. F. Lopez A. M. Barton B. A. (2020). Timing of initiation and duration of feeding rumen-protected choline affects performance of lactating Holstein cows. *J. Dairy Sci.* 103 4174–4191. 10.3168/jds.2019-17293 32171515

[B8] Cani P. D. Amar J. Iglesias M. A. Poggi M. Knauf C. Bastelica D. (2007). Metabolic endotoxemia initiates obesity and insulin resistance. *Diabetes* 56 1761–1772. 17456850 10.2337/db06-1491

[B9] Chen C. Fang S. Wei H. He M. Fu H. Xiong X. (2021). Prevotella copri increases fat accumulation in pigs fed with formula diets. *Microbiome* 9:175. 10.1186/s40168-021-01110-0 34419147 PMC8380364

[B10] Chen L. H. Huang S. Y. Huang K. C. Hsu C. C. Yang K. C. Li L. A. (2019). Lactobacillus paracasei PS23 decelerated age-related muscle loss by ensuring mitochondrial function in SAMP8 mice. *Aging* 11 756–770. 10.18632/aging.101782 30696799 PMC6366975

[B11] Chen Y. M. Wei L. Chiu Y. S. Hsu Y. J. Tsai T. Y. Wang M. F. (2016). Lactobacillus plantarum TWK10 Supplementation Improves Exercise Performance and Increases Muscle Mass in Mice. *Nutrients* 8:205. 10.3390/nu8040205 27070637 PMC4848674

[B12] Chen Z. Wong P. Y. Ng C. Lan L. Fung S. Li J. W. (2020). The Intersection between Oral Microbiota, Host Gene Methylation and Patient Outcomes in Head and Neck Squamous Cell Carcinoma. *Cancers* 12:3425. 10.3390/cancers12113425 33218162 PMC7698865

[B13] Cucchiara S. Stronati L. Aloi M. (2012). Interactions between intestinal microbiota and innate immune system in pediatric inflammatory bowel disease. *J. Clin. Gastroenterol.* 46 S64–S66. 22955361 10.1097/MCG.0b013e31826a857f

[B14] Dayama G. Priya S. Niccum D. E. Khoruts A. Blekhman R. (2020). Interactions between the gut microbiome and host gene regulation in cystic fibrosis. *Genome Med.* 12:12. 10.1186/s13073-020-0710-2 31992345 PMC6988342

[B15] den Besten G. Van Eunen K. Groen A. K. Venema K. Reijngoud D. J. Bakker B. M. (2013). The role of short-chain fatty acids in the interplay between diet, gut microbiota, and host energy metabolism. *J. Lipid. Res.* 54 2325–2340. 23821742 10.1194/jlr.R036012 PMC3735932

[B16] Donaldson G. P. Chou W. C. Manson A. L. Rogov P. Abeel T. Bochicchio J. (2020). Spatially distinct physiology of *Bacteroides* fragilis within the proximal colon of gnotobiotic mice. *Nat. Microbiol.* 5 746–756. 10.1038/s41564-020-0683-3 32152589 PMC7426998

[B17] Fancher C. A. Zhang L. Kiess A. S. Adhikari P. A. Dinh T. Sukumaran A. T. (2020). Avian Pathogenic *Escherichia coli* and Clostridium perfringens: Challenges in No Antibiotics Ever Broiler Production and Potential Solutions. *Microorganisms* 8:1533. 10.3390/microorganisms8101533 33036173 PMC7599686

[B18] Fang C. Zhong H. Lin Y. Chen B. Han M. Ren H. (2018). Assessment of the cPAS-based BGISEQ-500 platform for metagenomic sequencing. *Gigascience* 7 1–8. 10.1093/gigascience/gix133 29293960 PMC5848809

[B19] Franzosa E. A. Mciver L. J. Rahnavard G. Thompson L. R. Schirmer M. Weingart G. (2018). Species-level functional profiling of metagenomes and metatranscriptomes. *Nat. Methods* 15 962–968. 10.1038/s41592-018-0176-y 30377376 PMC6235447

[B20] Fung T. C. Vuong H. E. Luna C. Pronovost G. N. Aleksandrova A. A. Riley N. G. (2019). Intestinal serotonin and fluoxetine exposure modulate bacterial colonization in the gut. *Nat. Microbiol.* 4 2064–2073. 10.1038/s41564-019-0540-4 31477894 PMC6879823

[B21] Goodarzi P. Habibi M. Roberts K. Sutton J. Shili C. N. Lin D. (2021). Dietary Tryptophan Supplementation Alters Fat and Glucose Metabolism in a Low-Birthweight Piglet Model. *Nutrients* 13:2561. 10.3390/nu13082561 34444719 PMC8399558

[B22] He J. Zhang P. Shen L. Niu L. Tan Y. Chen L. (2020). “Short-Chain Fatty Acids and Their Association with Signalling Pathways in Inflammation. Glucose and Lipid Metabolism. *Int. J. Mol. Sci.* 21:6365. 10.3390/ijms21176356 PMC7503625 32887215

[B23] Hoffman J. M. Margolis K. G. (2020). Building community in the gut: a role for mucosal serotonin. *Nat. Rev. Gastroenterol. Hepatol.* 17 6–8. 10.1038/s41575-019-0227-6 31624372 PMC6930332

[B24] Huang W. C. Hsu Y. J. Huang C. C. Liu H. C. Lee M. C. (2020). Exercise Training Combined with Bifidobacterium longum OLP-01 Supplementation Improves Exercise Physiological Adaption and Performance. *Nutrients* 12:1145. 10.3390/nu12041145 32325851 PMC7231274

[B25] Jiao A. Yu B. He J. Yu J. Zheng P. Luo Y. (2021). Sodium acetate, propionate, and butyrate reduce fat accumulation in mice via modulating appetite and relevant genes. *Nutrition* 87–88:111198. 10.1016/j.nut.2021.111198 33761444

[B26] Kim D. Pertea G. Trapnell C. Pimentel H. Kelley R. Salzberg S. L. (2013). Tophat2: accurate alignment of transcriptomes in the presence of insertions, deletions and gene fusions. *Genome Biol.* 14:R36. 10.1186/gb-2013-14-4-r36 23618408 PMC4053844

[B27] Lahiri S. Kim H. Garcia-Perez I. Reza M. M. Martin K. A. Kundu P. (2019). The gut microbiota influences skeletal muscle mass and function in mice. *Sci. Transl. Med.* 11 eaan5662. 10.1126/scitranslmed.aan5662 31341063 PMC7501733

[B28] Lee E. Jung S. R. Lee S. Y. Lee N. K. Paik H. D. Lim S. I. (2018). “Lactobacillus plantarum Strain Ln4 Attenuates Diet-Induced Obesity, Insulin Resistance, and Changes in Hepatic mRNA Levels Associated with Glucose and Lipid Metabolism”. *Nutrients* 10:643. 10.3390/nu10050643 29783731 PMC5986522

[B29] Lee M. C. Hsu Y. J. Ho H. H. Hsieh S. H. Kuo Y. W. Sung H. C. (2020). Lactobacillus salivarius Subspecies salicinius SA-03 is a New Probiotic Capable of Enhancing Exercise Performance and Decreasing Fatigue. *Microorganisms* 8:545. 10.3390/microorganisms8040545 32283729 PMC7232535

[B30] Ley R. E. Turnbaugh P. J. Klein S. Gordon J. I. (2006). Microbial ecology: human gut microbes associated with obesity. *Nature* 444 1022–1023. 10.1038/4441022a 17183309

[B31] Li Y. Cheng G. Yamada T. Liu J. Zan L. Tong B. (2020). Effect of Expressions and SNPs of Candidate Genes on Intramuscular Fat Content in Qinchuan Cattle. *Animals* 10:1370. 10.3390/ani10081370 32784655 PMC7459438

[B32] Li Y. F. Jin W. Cheng Y. F. Zhu W. (2016). Effect of the associated methanogen *Methanobrevibacter thaueri* on the dynamic profile of end and intermediate metabolites of anaerobic fungus *Piromyces* sp. F1. *Curr. Microbiol.* 73 434–441. 10.1007/s00284-016-1078-9 27287262

[B33] Liu C. Cheung W. H. Li J. Chow S. K. Yu J. Wong S. H. (2021). Understanding the gut microbiota and sarcopenia: a systematic review. *J. Cachexia Sarcopenia Muscle* 12 1393–1407. 10.1002/jcsm.12784 34523250 PMC8718038

[B34] Liu L. Liu X. Cui H. Liu R. Zhao G. Wen J. (2019). Transcriptional insights into key genes and pathways controlling muscle lipid metabolism in broiler chickens. *BMC Genomics* 20:863. 10.1186/s12864-019-6221-0 31729950 PMC6858653

[B35] Lopez-Almela I. Romani-Perez M. Bullich-Vilarrubias C. Benitez-Paez A. Gomez D. P. E. Frances R. (2021). *Bacteroides* uniformis combined with fiber amplifies metabolic and immune benefits in obese mice. *Gut Microbes* 13 1–20. 10.1080/19490976.2020.1865706 33499721 PMC8018257

[B36] McNelis J. C. Lee Y. S. Mayoral R. Van Der Kant R. Johnson A. M. Wollam J. (2015). GPR43 Potentiates beta-Cell Function in Obesity. *Diabetes* 64 3203–3217. 10.2337/db14-1938 26023106 PMC4542437

[B37] Meng Q. Sun S. Sun Y. Li J. Wu D. Shan A. (2018). Effects of dietary lecithin and l-carnitine on fatty acid composition and lipid-metabolic genes expression in subcutaneous fat and longissimus thoracis of growing-finishing pigs. *Meat. Sci.* 136 68–78. 10.1016/j.meatsci.2017.10.012 29096289

[B38] Mlodzik-Czyzewska M. A. Malinowska A. M. Chmurzynska A. (2020). Low folate intake and serum levels are associated with higher body mass index and abdominal fat accumulation: a case control study. *Nutr. J.* 19:53. 32498709 10.1186/s12937-020-00572-6 PMC7273685

[B39] Nay K. Jollet M. Goustard B. Baati N. Vernus B. Pontones M. (2019). Gut bacteria are critical for optimal muscle function: a potential link with glucose homeostasis. *Am. J. Physiol. Endocrinol. Metab.* 317 E158–E171. 10.1152/ajpendo.00521.2018 31039010

[B40] Nguyen T. Sedghi L. Ganther S. Malone E. Kamarajan P. Kapila Y. L. (2020). Host-microbe interactions: Profiles in the transcriptome, the proteome, and the metabolome. *Periodontol* 2000 115–128. 10.1111/prd.12316 31850641 PMC7968888

[B41] Ni Y. Yang X. Zheng L. Wang Z. Wu L. Jiang J. (2019). Lactobacillus and Bifidobacterium Improves Physiological Function and Cognitive Ability in Aged Mice by the Regulation of Gut Microbiota. *Mol. Nutr. Food Res.* 63:e1900603. 10.1002/mnfr.201900603 31433910

[B42] Nordhoff M. Taras D. Macha M. Tedin K. Busse H. J. Wieler L. H. (2005). Treponema berlinense sp. nov. and Treponema porcinum sp. nov., novel spirochaetes isolated from porcine faeces. *Int. J. Syst. Evol. Microbiol.* 55 1675–1680. 10.1099/ijs.0.63388-0 16014500

[B43] Nyholm L. Koziol A. Marcos S. Botnen A. B. Aizpurua O. Gopalakrishnan S. (2020). Holo-Omics: Integrated Host-Microbiota Multi-omics for Basic and Applied Biological Research. *Iscience* 23:101414. 10.1016/j.isci.2020.101414 32777774 PMC7416341

[B44] Parseus A. Sommer N. Sommer F. Caesar R. Molinaro A. Stahlman M. (2017). Microbiota-induced obesity requires farnesoid X receptor. *Gut* 66 429–437. 10.1136/gutjnl-2015-310283 26740296 PMC5534765

[B45] Paster B. J. Canale-Parola E. (1985). Treponema saccharophilum sp. nov., a large pectinolytic spirochete from the bovine rumen. *Appl. Environ. Microbiol.* 50 212–219. 10.1128/aem.50.2.212-219.1985 4051480 PMC238606

[B46] Qi K. Men X. Wu J. Xu Z. (2019). Rearing pattern alters porcine myofiber type, fat deposition, associated microbial communities and functional capacity. *BMC Microbiol.* 19:181. 10.1186/s12866-019-1556-x 31387544 PMC6683424

[B47] Qian Y. Li M. Wang W. Wang H. Zhang Y. Hu Q. (2019). Effects of Lactobacillus Casei YBJ02 on Lipid Metabolism in Hyperlipidemic Mice. *J. Food Sci.* 84 3793–3803. 10.1111/1750-3841.14787 31762034

[B48] Qin J. Li Y. Cai Z. Li S. Zhu J. Zhang F. (2012). A metagenome-wide association study of gut microbiota in type 2 diabetes. *Nature* 490 55–60. 10.1038/nature11450 23023125

[B49] Ryan M. P. Adley C. C. (2010). Sphingomonas paucimobilis: a persistent Gram-negative nosocomial infectious organism. *J. Hosp. Infect.* 75 153–157. 10.1016/j.jhin.2010.03.007 20434794

[B50] Salaj R. Stofilova J. Soltesova A. Hertelyova Z. Hijova E. Bertkova I. (2013). The effects of two Lactobacillus plantarum strains on rat lipid metabolism receiving a high fat diet. *ScientificWorldJournal* 2013:135142. 10.1155/2013/135142 PMC3891428 24470789

[B51] Schoeler M. Caesar R. (2019). Dietary lipids, gut microbiota and lipid metabolism. *Rev. Endocr. Metab. Disord.* 20 461–472. 10.1007/s11154-019-09512-0 31707624 PMC6938793

[B52] Shi F. Zi Y. Lu Z. Li F. Yang M. Zhan F. (2020). Bacillus subtilis H2 modulates immune response, fat metabolism and bacterial flora in the gut of grass carp (Ctenopharyngodon idellus). *Fish. Shellfish Immunol.* 106 8–20. 10.1016/j.fsi.2020.06.061 32717323

[B53] Siddharth J. Chakrabarti A. Pannerec A. Karaz S. Morin-Rivron D. Masoodi M. (2017). Aging and sarcopenia associate with specific interactions between gut microbes, serum biomarkers and host physiology in rats. *Aging* 9 1698–1720. 10.18632/aging.101262 28783713 PMC5559170

[B54] Stojanov S. Berlec A. Strukelj B. (2020). The Influence of Probiotics on the Firmicutes/Bacteroidetes Ratio in the Treatment of Obesity and Inflammatory Bowel disease. *Microorganisms* 8:1715. 10.3390/microorganisms8111715 33139627 PMC7692443

[B55] Tanner S. A. Chassard C. Rigozzi E. Lacroix C. Stevens M. T. A. (2016). *Bifidobacterium thermophilum* RBL67 impacts on growth and virulence gene expression of *Salmonella enterica* subsp. *enterica* serovar Typhimurium. *BMC Microbiol.* 16:46. 10.1186/s12866-016-0659-x 26988691 PMC4797131

[B56] Tanner S. A. Lacroix C. Del’Homme C. Jans C. Berner A. Z. Bernalier-Donadille A. (2015). Effect of *Bifidobacterium thermophilum* RBL67 and fructo-oligosaccharides on the gut microbiota in Göttingen minipigs. *Br. J. Nutr.* 114 746–755. 10.1017/S0007114515002263 26313935

[B57] Toure R. Kheadr E. Lacroix C. Moroni O. Fliss I. (2003). Production of antibacterial substances by bifidobacterial isolates from infant stool active against *Listeria monocytogenes*. *J. Appl. Microbiol.* 95 1058–1069. 10.1046/j.1365-2672.2003.02085.x 14633035

[B58] Vagnerová K. Vodicka M. Hermanová P. Ergang P. Šrutková D. Klusonová P. (2019). Interactions Between Gut Microbiota and Acute Restraint Stress in Peripheral Structures of the Hypothalamic-Pituitary-Adrenal Axis and the Intestine of Male Mice. *Front. Immunol.* 10:2655. 10.3389/fimmu.2019.02655 31798585 PMC6878942

[B59] Verhulst N. O. Takken W. Dicke M. Schraa G. Smallegange R. C. (2010). Chemical ecology of interactions between human skin microbiota and mosquitoes. *FEMS Microbiol. Ecol.* 74 1–9. 20840217 10.1111/j.1574-6941.2010.00908.x

[B60] Wang J. Chen L. Zhao N. Xu X. Xu Y. Zhu B. (2018). Of genes and microbes: solving the intricacies in host genomes. *Protein Cell.* 9 446–461. 10.1007/s13238-018-0532-9 29611114 PMC5960464

[B61] Wen C. Yan W. Sun C. Ji C. Zhou Q. Zhang D. (2019). The gut microbiota is largely independent of host genetics in regulating fat deposition in chickens. *ISME J.* 13 1422–1436. 10.1038/s41396-019-0367-2 30728470 PMC6775986

[B62] Won M. Y. Oyama L. B. Courtney S. J. Creevey C. J. Huws S. A. (2020). Can rumen bacteria communicate to each other? *Microbiome* 8:23. 10.1186/s40168-020-00796-y 32085816 PMC7035670

[B63] Xavier R. J. Podolsky D. K. (2007). Unravelling the pathogenesis of inflammatory bowel disease. *Nature* 448 427–434. 10.1038/nature06005 17653185

[B64] Xie N. Cui Y. Yin Y. N. Zhao X. Yang J. W. Wang Z. G. (2011). Effects of two Lactobacillus strains on lipid metabolism and intestinal microflora in rats fed a high-cholesterol diet. *BMC Complement Altern. Med.* 11:53. 10.1186/1472-6882-11-53 21722398 PMC3144010

[B65] Xue M. Sun H. Wu X. Guan L. L. Liu J. (2018). Assessment of Rumen Microbiota from a Large Dairy Cattle Cohort Reveals the Pan and Core Bacteriomes Contributing to Varied Phenotypes. *Appl. Environ. Microbiol.* 84 e970–18. 10.1128/AEM.00970-18 30054362 PMC6146982

[B66] Yan H. Diao H. Xiao Y. Li W. Yu B. He J. (2016). Gut microbiota can transfer fiber characteristics and lipid metabolic profiles of skeletal muscle from pigs to germ-free mice. *Sci. Rep.* 6:31786. 10.1038/srep31786 27545196 PMC4992887

[B67] Zambell K. L. Fitch M. D. Fleming S. E. (2003). Acetate and butyrate are the major substrates for de novo lipogenesis in rat colonic epithelial cells. *J. Nutr.* 133 3509–3515. 10.1093/jn/133.11.3509 14608066

[B68] Zelante T. Iannitti R. G. Cunha C. De Luca A. Giovannini G. Pieraccini G. (2013). Tryptophan catabolites from microbiota engage aryl hydrocarbon receptor and balance mucosal reactivity via interleukin-22. *Immunity* 39 372–385. 10.1016/j.immuni.2013.08.003 23973224

[B69] Zheng D. Liwinski T. Elinav E. (2020). Interaction between microbiota and immunity in health and disease. *Cell Res.* 30 492–506. 10.1038/s41422-020-0332-7 32433595 PMC7264227

