# Supplementary material for: Metagenomic and Transcriptomic Analyses Reveal the Differences and Associations Between the Gut Microbiome and Muscular Genes in Angus and Chinese Simmental Cattle
Source: Front Microbiol. 2022 Apr 5;13:815915. doi: 10.3389/fmicb.2022.815915 (PMC9048903; doi:10.3389/fmicb.2022.815915)

Supplementary materials for the "**Metagenomic and transcriptomic analyses reveal the differences and associations between the gut microbiome and muscular genes in Angus and Chinese Simmental cattle**"

The manuscript including 8 supplementary figures and 2 supplementary tables. The details were shown as below:

Table S1. Differences in meat quality related indicators of the Angus and Chinese Simmental cattle.

	**Angus**	**Chinese Simmental**	***P* value**
Live weight (kg)	713.36 ± 41.35	687.18 ± 33.86	N.D.
carcass weight (kg)	395.78 ± 24.72	404.87 ± 20.23	N.D.
Crude fat (g/100g)	4.62 ± 0.25	4.62 ± 0.25	< 0.01
Types of fatty acid	17	15	-
Types of saturated fatty acid	5	5	-
Types of unsaturated fatty acid	12	10	-
Total saturated fatty acid (%)	35.685 ± 3.045	42.885 ± 2.559	< 0.05
Total unsaturated fatty acids (%)	64.315 ± 3.045	57.115 ± 2.559	< 0.01
Total monounsaturated fatty acids (%)	58.620 ± 2.517	49.694 ± 2.444	< 0.05
Total polyunsaturated fatty acids (%)	5.695 ± 0.755	7.421 ± 2.607	< 0.05

Table S2. Table S2 including several worksheet so we have uploaded as a seperate file.

Figure S1. The intramuscular fat of the Angus (a) and Chinese Simmental (b) cattle is significantly different.

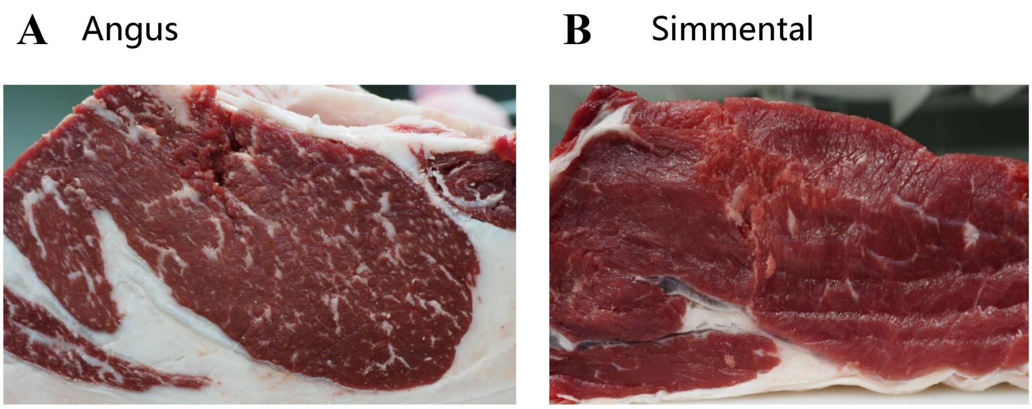

Figure S2. The beta-diversity (a) and alpha-diversity (b) at species level, and the top 5 abundant phyla (c) for the two breeds. (Supplementary figures for Figure 1)

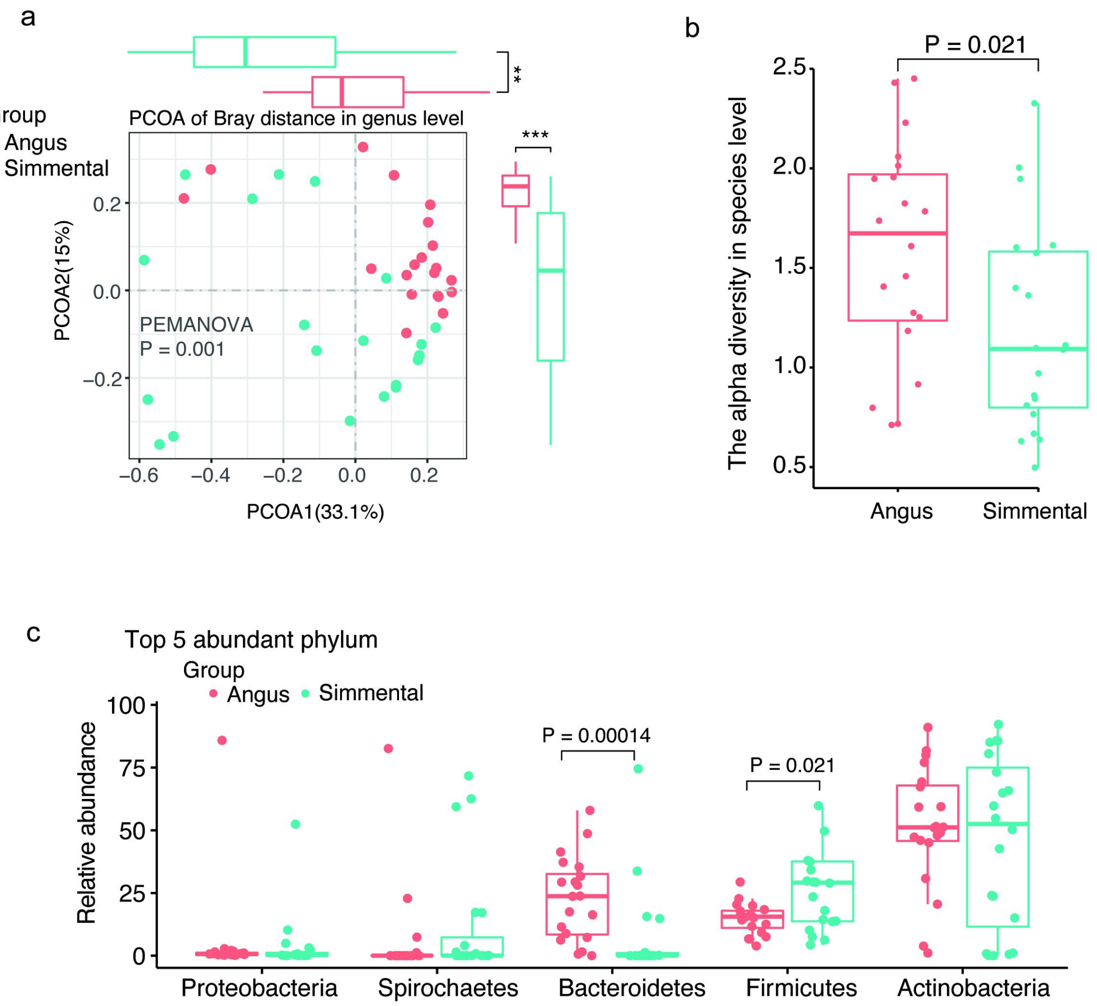

Figure S3. The species that were significantly related to superpathway of fatty acid biosynthesis initiation. (Supplementary figures for Figure 3)

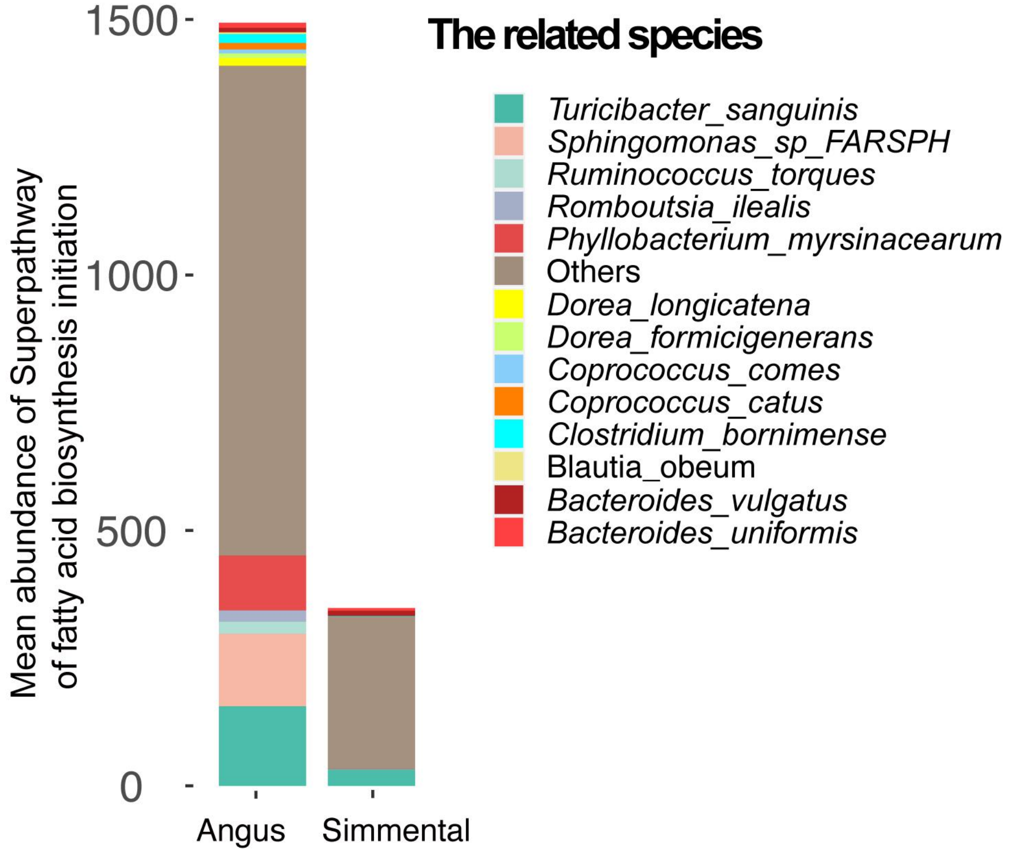

Figure S4. The differences of short chain fatty acids for the two breeds.

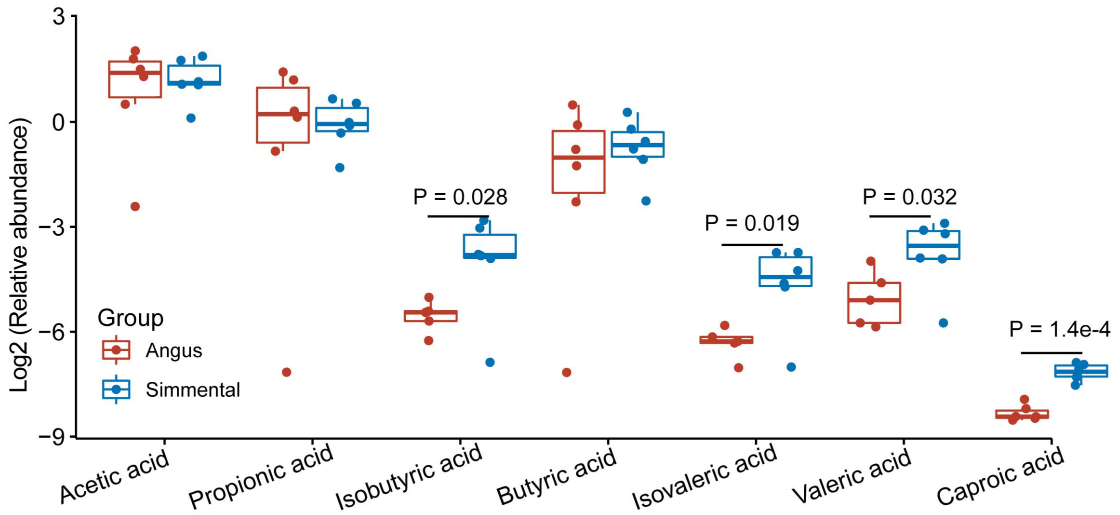

Figure S5. Functional enrichment at molecular function (a), biological process (b), cellular component (c) and KEEG (d) of all the DEGs between the two breeds. (Supplementary figures for Figure 1b and 1c).

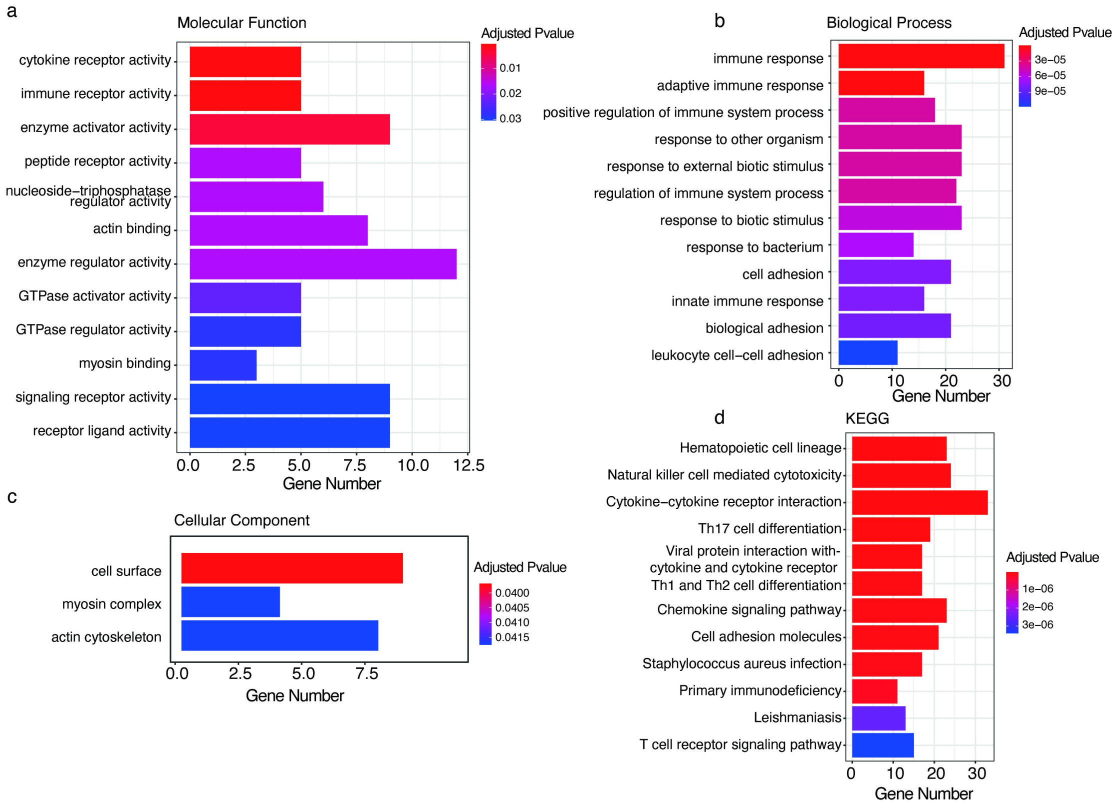

Figure S6. The PCA of the DEGs between the Angus and Chinese Simmental cattle.

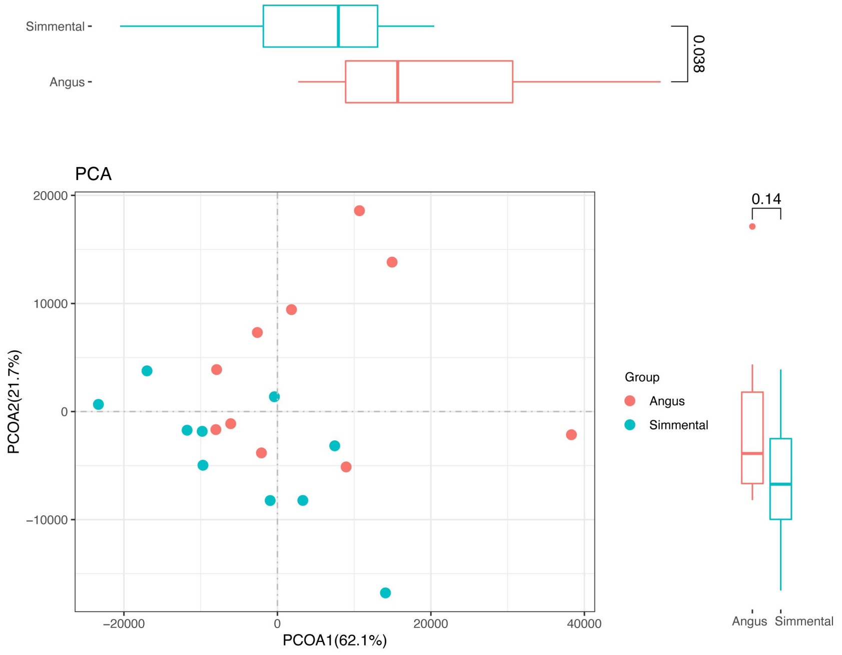

Figure S7. Correlation between the SCFAs and differentially enriched species between the two groups.

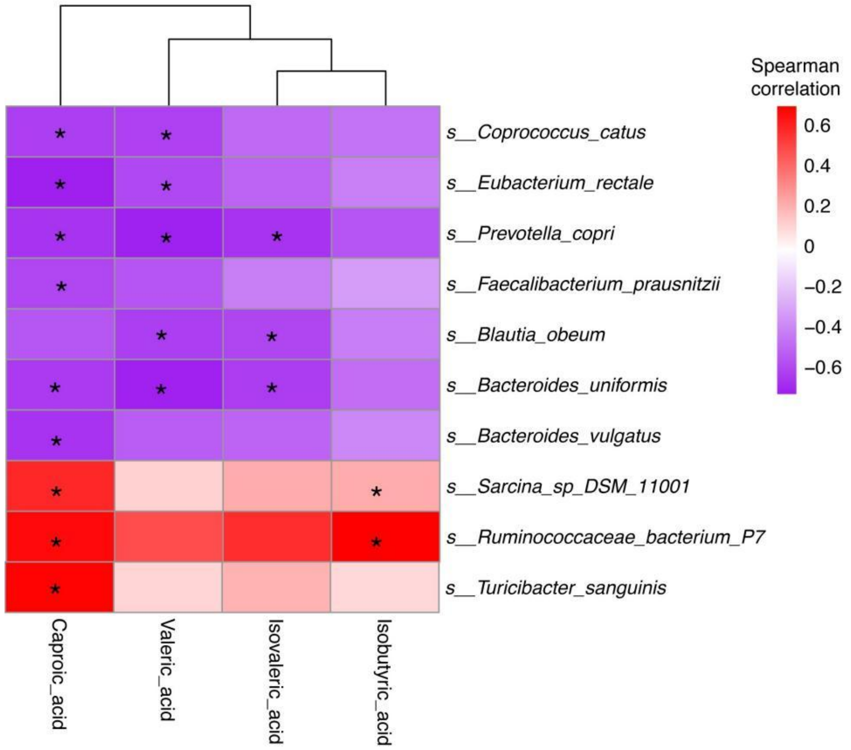

Figure S8. Correlation between the SCFAs and the top 3 DEGs between the two groups.

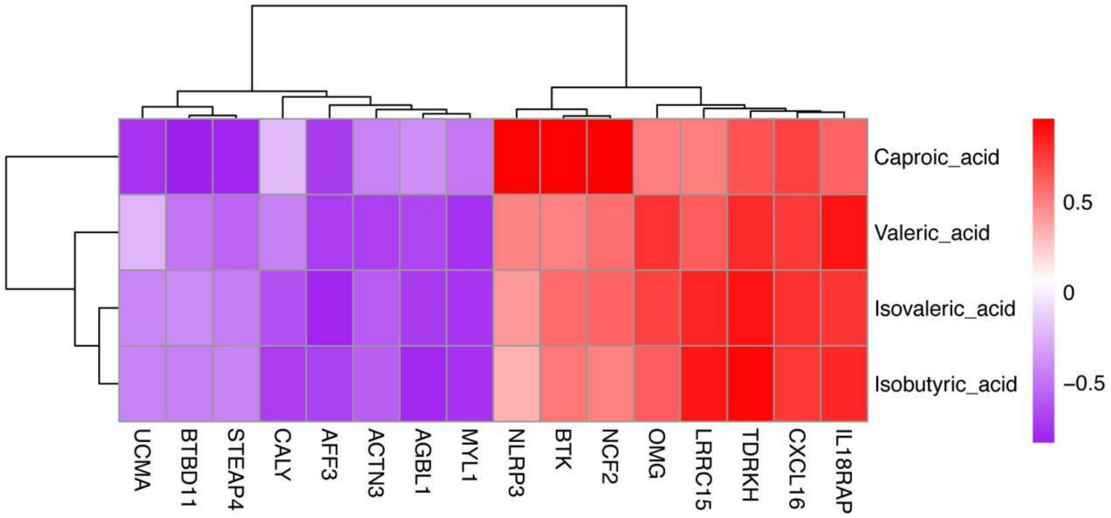

Supplement: Supplementary file 1 [file Presentation_1.pdf]
